# Nonlinear relationship with saturation effect observed between neutrophil to high-density lipoprotein cholesterol ratio and atherosclerosis in a health examination population: a cross‐sectional study

**DOI:** 10.1186/s12872-022-02869-2

**Published:** 2022-09-26

**Authors:** Yaqing Zhou, Haijun Dan, Long Bai, Limei Jia, Baojin Lu, Wei Cui

**Affiliations:** 1 Department of Cardiology, The Second Hospital of Hebei Medical University and Institute of Cardiocerebrovascular Disease of Hebei Province, No. 215, He Ping West Road, Shijiazhuang, 050000 China; 2 Department of Physical Examination Center, The Second Hospital of Hebei Medical University, Shijiazhuang, 050000 China

**Keywords:** Atherosclerosis, Neutrophil to high-density lipoprotein cholesterol ratio, Brachial-ankle pulse wave velocity, NHR, Ba-PWV

## Abstract

**Background:**

The relationships between inflammatory indexes and atherosclerosis as well as those between blood lipid indexes and atherosclerosis have been widely studied, but the relationship between the neutrophil to high-density lipoprotein cholesterol ratio (NHR) and atherosclerosis had not been investigated until the present study.

**Methods:**

For this cross‐sectional study, we continuously collected data from a health examination population in the Second Hospital of Hebei Medical University from January 2012 to December 2017 (N = 1978). The collected data included clinical data, hematological indexes, and brachial-ankle pulse wave velocity (Ba-PWV). Atherosclerosis was defined as Ba-PWV ≥ 1400 cm/s. The relationship between the NHR and atherosclerosis was explored via univariate regression analysis, multivariate regression analysis, smoothing function analysis, and analysis of a threshold saturation effect.

**Results:**

Among 1978 participants, the mean age was 54 years, 1189 participants (60.11%) were male, and 1103 (55.76%) had a history of atherosclerosis. Univariate analysis showed a positive association between the NHR and atherosclerosis [odds ratio (OR) = 1.19, 95% confidence interval (CI): 1.11–1.27, *P* < 0.01], and this positive association remained significant on multivariate analyses with adjustments for confounding factors (OR = 1.14, 95% CI: 1.06–1.24, *P* < 0.01). Generalized additive model results revealed a non-linear relationship with a saturation effect between the NHR and atherosclerosis, with a threshold at 3.32. At values ≤ 3.32, the NHR was positively associated with atherosclerosis, but the association was not statistically significant for values > 3.32.

**Conclusion:**

A nonlinear relationship with a certain saturation effect was observed between the NHR and atherosclerosis in a health examination population.

## Background

Atherosclerosis is a common pathological contributor to various cardiovascular and cerebrovascular diseases, and research has demonstrated the value of atherosclerosis for assessing the risk, evaluating the severity, and predicting the prognosis of cardiovascular and cerebrovascular diseases [1, 2]. Atherosclerosis was also shown to be an independent risk factor for cognitive decline [3]. Furthermore, quantification of atherosclerosis as a continuous variable in the form of pulse wave velocity (PWV) has been recognized as a good indicator of vascular aging [4] and able to distinguish patients with healthy versus accelerated vascular aging [5]. Therefore, early detection and effective intervention in patients with atherosclerosis is important for delaying and preventing the development of cardiovascular and cerebrovascular diseases and their complications. The brachial-ankle pulse wave velocity (Ba-PWV) is a parameter used to evaluate the elasticity of arteries and an index commonly used for early diagnosis of atherosclerosis. The Ba-PWV is proportional to the degree of atherosclerosis and strongly associated with cardiovascular and cerebrovascular events and all-cause mortality. The present study used Ba-PWV to assess atherosclerosis [2, 6–8]. The normal range of Ba-PWV is commonly defined as < 1400 cm/s and atherosclerosis is defined by a Ba-PWV ≥ 1400 cm/s [9–12].

At present, it is widely believed that chronic vascular inflammation and hyperlipidemia are the key factors for the development of atherosclerosis [13–23]. Changes in physiological status, such as hyperlipidemia, increased circulating levels of pro-inflammatory cytokines, and high shear stress, can cause endothelial dysfunction, which can initiate the development of atherosclerosis [20–23]. The entry of oxidized low-density lipoprotein cholesterol (LDL-C) into the endothelium further exacerbates the damage to endothelial function and leads to leukocyte recruitment. Leukocytes then further release growth factors and inflammatory mediators, aggravating the chronic inflammatory response within blood vessels [22, 24]. Long-term inflammation then leads to vascular smooth muscle proliferation, microvascular formation, and subsequent atherosclerosis [25–27].

The neutrophil to high-density lipoprotein cholesterol ratio (NHR) is a recently discovered marker [28] that combines indicators of inflammation and hyperlipidemia. Neutrophils can promote the inflammatory response and oxidative stress, further aggravating atherosclerosis [29], whereas high-density lipoprotein cholesterol (HDL-C) can improve endothelial function by inhibiting the inflammatory response and oxidative stress, thereby protecting blood vessels [30]. The neutrophil count and serum HDL-C level in combination form a dynamic index that can more fully represent the inflammatory response and oxidative stress of the body than either individual parameter. To date, research on the NHR has mainly focused on its use for the prognosis of cardiovascular and cerebrovascular diseases [31–34]. The NHR was shown to be valuable for assessing the severity and predicting the outcome of coronary artery disease [31, 32]. The NHR was also proven to be associated with poor outcomes in acute ischemic stroke patients [33]. Atherosclerosis is the foundation of early cardiovascular and cerebrovascular diseases. However, the relationship between the NHR and early atherosclerosis has not been established.

The present study comprehensively assessed the association between the NHR, which combines inflammatory and blood lipid indexes, and atherosclerosis. Moreover, we use the generalized additive model to explore the potential nonlinear relationship in order to evaluate the relationship accurately and quantitatively. Further, a health examination population was chosen as the study population, because no related study in this particular population has been reported. Therefore, this study assessed the relationship between the NHR and atherosclerosis in a health examination population with strict control of confounding factors. Furthermore, the potential nonlinear relationship was explored.

## Methods

### Study population

**Fig. 1:**
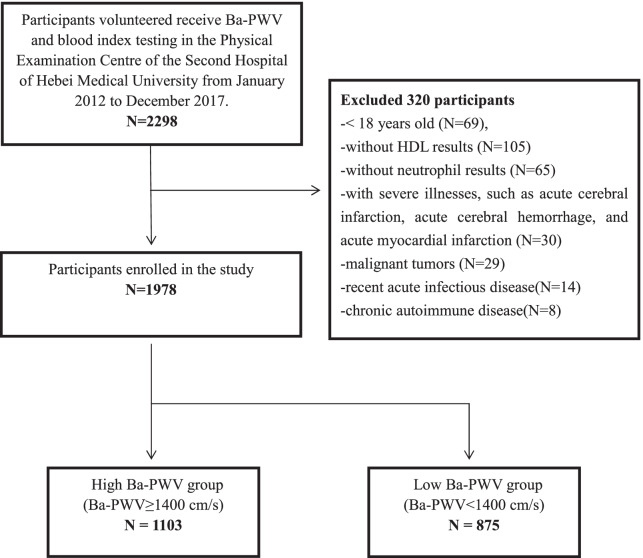
A schematic illustrating the inclusion/exclusion of the participants

In this study, a total of 2298 adults who underwent physical examination in the Physical Examination Center of the Second Hospital of Hebei Medical University were recruited from January 2012 to December 2017. Of these, 320 participants were excluded from the study for data missing or the presence of severe diseases. The final study included 1978 participants (Fig. 1). This study was approved by the Ethics Committee of the Second Hospital of Hebei Medical University. All participants informed consent and volunteered to participate in the study.

The eligibility criteria included having received Ba-PWV testing and blood index testing at the same time. The exclusion criteria included: (1) recent severe illness including acute coronary heart disease and cerebrovascular disease; (2) recent acute or chronic infectious disease or chronic autoimmune disease; (3) malignant tumors or blood system diseases; and (4) incomplete records from physical examination.

### Data collection

Physical examination data for all participants were collected from the Physical Examination Centre of the Second Hospital of Hebei Medical University, including general clinical data [name, sex, age, height, weight, systolic blood pressure, diastolic blood pressure, heart rate (HR), etc.], treatment-related clinical data (history of hypertension, diabetes, smoking and alcohol consumption), hematological indexes (routine blood test results, liver and kidney function test results, blood lipid levels, etc.), and Ba-PWV.

### Diagnostic criteria and grouping methods

Ba-PWV is defined as the speed at which the pulse wave propagates between the brachial artery and the ankle artery. With increasing pulse wave conduction velocity, the elasticity of the artery is reduced and the degree of arteriosclerosis is higher. The normal range of Ba-PWV is defined as < 1400 cm/s, and atherosclerosis is defined by Ba-PWV ≥ 1400 cm/s [9–12].

A Ba-PWV cut-off value of ≥ 1400 cm/s was shown to be useful for screening for cardiovascular risk and the severity of atherosclerosis in the general population [35]. Ba-PWV was measured using the Omron atherosclerotic apparatus (BP-203 RPE III). For Ba-PWV measurement, the patient lay flat on the examination bed and remained in a resting state for about 5 min. Blood pressure cuffs were attached over the brachial artery on the medial parts of the legs, and electrodes were placed at the wrists for electrocardiogram (ECG) monitoring. Sensors were placed between the left side of the sternum and the fourth intercostal space to monitor heart sounds. During this process, the pulse wave was recorded, and the PWV on both sides was calculated automatically by the instrument, according to the distance and time of the conduction pulse wave. Higher Ba-PWV values indicate worsening atherosclerosis.

Neutrophil count and HDL-C level were measured under fasting conditions in samples of 5 ml of venous blood taken between 7 and 9 am. The blood samples were centrifuged for 10 min at 1912 × *g* in a BY-600A centrifuge (Beijing Baiyang Medical Instrument Co., Ltd.), and serum was extracted. After centrifugation, the serum HDL-C concentration was analyzed using an Automatic Biochemical Analyzer (Beckman Coulter AU5800). The neutrophil count was determined by a blood cell analyzer (UniCel DxH 800, Beckman Coulter).

Blood pressure was measured using an electronic sphygmomanometer (Omron HBP‐9012; OMRON Healthcare Product Development Dalian Co., Ltd.). The electronic sphygmomanometer has undergone strict quality control and meets regulatory requirements and accuracy standards for BP measuring devices [36]. Three consecutive measurements of blood pressure were performed, with an interval of 1 to 2 min between measurements. The average of the second and third measurements was used as the final BP.

### Statistical analysis

Continuous variables were described as mean ± standard deviation (SD) for a normal distribution and median with inter-quartile-range (IQR) values for a skewed distribution, while the classification variables were described as percentages (%). We used one-way analysis of variance (ANOVA) for normally continuous variables, the non-parametric test for skewed continuous variables and the chi-square test (χ^2^) and Fisher exact probability method for classification variables. The relationship between the NHR and atherosclerosis was analyzed by univariate and multivariate regression analysis. Multivariate regression analysis included gradual adjustment for confounding factors. According to the recommendation of the STROBE statement, we simultaneously produced results from unadjusted, minimally adjusted and fully adjusted analyses. The unadjusted model included adjustment for no variables. The minimally adjusted model (Model I) included adjustment for sex, age group and the fully adjusted model (Model II) included adjustment for sex, age group, body mass index (BMI) group, HR, LDL-C group, drinking, smoking, hypertension and diabetes. The odd ratio (OR) and 95% confidence interval (95%CI) were calculated for the associations between NHR (per-1 unit increase and per-SD increase) and atherosclerosis. Then, the NHR was divided by quartiles (25%, 50%, and 75%) as follows: Q1 (< 2.04 × 10^9^/mmol), Q2 (≥ 2.04 ~ < 2.72 × 10^9^/mmol), Q3 (≥ 2.72 ~ < 3.64 × 10^9^/mmol), and Q4 (≥ 3.64 × 10^9^/mmol). We performed testing for a linear trend by entering the median value of each category of NHR as a continuous variable in the models (sensitivity analysis).

We also conducted subgroup analyses to assess the relationship between the NHR and atherosclerosis according to different subgroups (sex subgroups: female and male; age subgroups: ≤ 45, 45 ~ 65 and ≥ 65 years; BMI subgroups: < 18.5, ≥ 18.5 ~ < 24, ≥ 24 ~ < 28 and ≥ 28 kg/m^2^; LDL-C subgroups: < 2.07, ≥ 2.07 ~ ≤ 3.37 and > 3.37 mmol/L; drinking subgroups: no drinking and drinking; smoking subgroups: no smoking and smoking; diabetes subgroups: no diabetes and diabetes; and hypertension subgroups: no hypertension and hypertension). The interaction analysis was used to explore the consistency between different subgroups.

Finally, a smoothing function analysis of the generalized additive model (GAM) was applied to explore the potential nonlinear relationship between the NHR and atherosclerosis. After adjustment for confounding factors (sex, age group, BMI group, HR, LDL-C group, drinking, smoking, hypertension and diabetes), the relationship between the NHR and atherosclerosis was analyzed. The threshold saturation effect was applied to explore whether a threshold or saturation effect existed between the NHR and atherosclerosis. Values of *P* < 0.05 indicated statistical significance. All statistical analyses were performed using the statistical software R (http://www.R-project.org, The R Foundation) and Empower Stats (http://www.empowerstats.com, X&Y Solutions, Inc., Boston, MA).

## Results

### Demographic and clinical characteristics of participants stratified by NHR quartile

The demographic and clinical characteristics of the participants in the different NHR groups are presented in Table 1. Among 1978 participants, the mean age was 54 years, and 1189 participants (60.11%) were male. A total of 1103 (55.76%) had a history of atherosclerosis. Compared with that in the Q1 group, the Ba-PWV and prevalence of atherosclerosis were significantly higher in the Q4 group (*P* < 0.01). Additionally, there were significant differences among the NHR groups in BMI, HR, sex, smoking, drinking, hypertension and diabetes (*P* < 0.05; Table 1).

**Table 1:** Demographic and clinical characteristics of participants in the different NHR groups (N = 1978)

Characteristic	NHR				*P*
	Q1 (< 2.04)	Q2 (≥ 2.04 ~ < 2.72)	Q3 (≥ 2.72 ~ < 3.64)	Q4 (≥ 3.64)	
N	495	494	494	495	
Sex					< 0.01
Female	316 (63.84%)	217 (43.93%)	152 (30.77%)	104 (21.01%)	
Male	179 (36.16%)	277 (56.07%)	342 (69.23%)	391 (78.99%)	
Age groups					0.051
≤ 45	73 (14.75%)	76 (15.38%)	103 (20.85%)	99 (20.00%)	
(45 ~ 65)	337 (68.08%)	336 (68.02%)	321 (64.98%)	332 (67.07%)	
≥ 65	85 (17.17%)	82 (16.60%)	70 (14.17%)	64 (12.93%)	
BMI groups					< 0.01
< 18.5	17 (3.47%)	5 (1.01%)	7 (1.43%)	2 (0.41%)	
≥ 18.5 ~ < 24	235 (47.96%)	168 (34.08%)	132 (26.88%)	82 (16.67%)	
≥ 24 ~ < 28	182 (37.14%)	235 (47.67%)	214 (43.58%)	233 (47.36%)	
≥ 28	56 (11.43%)	85 (17.24%)	138 (28.11%)	175 (35.57%)	
NA	17 (3.43%)	14 (2.83%)	16 (3.24%)	16 (3.23%)	
HR (bpm)	73.48 ± 9.59	73.24 ± 9.88	75.24 ± 9.60	76.14 ± 9.96	< 0.01
Ba-PWV (cm/s)	**1426.57 ± 305.94**	**1465.32 ± 267.56**	**1486.29 ± 272.87**	**1513.57 ± 277.06**	**< 0.01**
Ba-PWV groups					**< 0.01**
< 1400	**269 (54.34%)**	**220 (44.53%)**	**199 (40.28%)**	**187 (37.78%)**	
≥ 1400	**226 (45.66%)**	**274 (55.47%)**	**295 (59.72%)**	**308 (62.22%)**	
LDL-C groups					0.087
< 2.07	46 (9.29%)	35 (7.09%)	37 (7.49%)	54 (10.91%)	
≥ 2.07 ~ ≤ 3.37	274 (55.35%)	246 (49.80%)	262 (53.04%)	256 (51.72%)	
> 3.37	175 (35.35%)	213 (43.12%)	195 (39.47%)	185 (37.37%)	
Drinking					**< **0.01
No	319 (64.44%)	252 (51.01%)	198 (40.08%)	208 (42.02%)	
Yes	130 (26.26%)	204 (41.30%)	232 (46.96%)	242 (48.89%)	
NA	46 (9.29%)	38 (7.69%)	64 (12.96%)	45 (9.09%)	
Smoking					**< **0.01
No	378 (76.36%)	341 (69.03%)	269 (54.45%)	220 (44.44%)	
Yes	71 (14.34%)	112 (22.67%)	158 (31.98%)	230 (46.46%)	
NA	46 (9.29%)	41 (8.30%)	67 (13.56%)	45 (9.09%)	
Diabetes history					0.013
No	429 (86.67%)	433 (87.65%)	407 (82.39%)	413 (83.43%)	
Yes	16 (3.23%)	19 (3.85%)	18 (3.64%)	32 (6.46%)	
NA	50 (10.10%)	42 (8.50%)	69 (13.97%)	50 (10.10%)	
Hypertension history				**< **0.01	
No	391 (78.99%)	379 (76.72%)	347 (70.24%)	346 (69.90%)	
Yes	54 (10.91%)	73 (14.78%)	78 (15.79%)	99 (20.00%)	
NA	50 (10.10%)	42 (8.50%)	69 (13.97%)	50 (10.10%)	

The bold values are used to highlight the change of the important indicator (Ba-PWV) in the different NHR groups

*Ba-PWV* Brachial-ankle pulse wave velocity; *BMI* Body mass index; *HDL-C* High density lipoprotein cholesterol; *NHR* Neutrophil to high-density lipoprotein cholesterol ratio; *LDL-C* Low-density lipoprotein cholesterol; *HR* Heart rate. *NA* Not available

### Positive association between the NHR and atherosclerosis

The NHR was positively associated with atherosclerosis without adjustment for confounding factors (OR = 1.19, 95% CI: 1.11–1.27, *P* < 0.01), and the positive association remained significant after adjustments for confounding factors in Model I (adjusted for sex and age group, Model I: OR = 1.20, 95% CI: 1.11–1.29, *P* < 0.01) and Model II (fully adjusted for sex, age group as well as BMI group, HR, LDL-C group, drinking, smoking, hypertension and diabetes, Model II: OR = 1.14, 95% CI: 1.06–1.24, *P* = 0.001). Moreover, each additional SD in NHR was associated with a 22% increase in the risk of atherosclerosis in fully adjusted model (OR = 1.22, 95% CI: 1.08–1.37, *P* = 0.001). When the NHR was presented in the form of a categorical variable, the risk of atherosclerosis in the Q4 group was increased 67% compared with that in the Q1 group (OR = 1.67, 95% CI: 1.22–2.29, *P* = 0.001). The positive relationship was also found in testing for a linear trend (OR = 1.19, 95% CI: 1.08–1.31, *P* = 0.001; Tables 2 and 3).

**Table 2:** Univariate analysis of potential associations between baseline characteristics and atherosclerosis

Covariate	Statistics	OR (95%CI)	*P*
Sex			
Female	789 (39.89%)	Reference	
Male	1189 (60.11%)	1.48 (1.24, 1.78)	**< **0.01
Age groups			
≤ 45	351 (17.75%)	Reference	
(45 ~ 65)	1326 (67.04%)	3.37 (2.60, 4.36)	**< **0.01
≥ 65	301 (15.22%)	16.61 (11.09, 24.87)	**< **0.01
HR (bpm)	74.55 ± 9.28	1.02 (1.01, 1.03)	0.000
Neutrophil (× 109/L)	**3.66 ± 1.24**	**1.29 (1.19, 1.40)**	**< 0.01**
HDL-C (mmol/L)	**1.32 ± 0.34**	**0.72 (0.55, 0.93)**	**0.013**
NHR (× 109/mmol)	**3.02 ± 1.48**	**1.19 (1.11, 1.27)**	**< 0.01**
NHR groups			
Q1 (< 2.04)	**495 (25.03%)**	**Reference**	
Q2 (≥ 2.04 ~ **< **2.72)	**494 (24.97%)**	**1.48 (1.15, 1.90)**	**0.002**
Q3 (≥ 2.72 ~ **< **3.64)	**494 (24.97%)**	**1.76 (1.37, 2.27)**	**< 0.01**
Q4 (≥ 3.64)	**495 (25.03%)**	**1.96 (1.52, 2.53)**	**< 0.01**
BMI groups			
** < **18.5	31 (1.57%)	Reference	
≥ 18.5 ~ **< **24	607 (30.69%)	1.14 (0.55, 2.36)	0.722
≥ 24 ~ **< **28	840 (42.47%)	1.62 (0.79, 3.33)	0.190
≥ 28	437 (22.09%)	2.08 (1.00, 4.34)	0.050
NA	63 (3.19%)	1.97 (0.83, 4.72)	0.126
LDL-C groups			
** < **2.07	172 (8.70%)	Reference	
≥ 2.07 ~ ≤ 3.37	1038 (52.48%)	1.22 (0.89, 1.69)	0.222
> 3.37	768 (38.83%)	1.88 (1.35, 2.63)	0.000
Drinking			
No	977 (49.39%)	Reference	
Yes	808 (40.85%)	1.24 (1.03, 1.50)	0.022
NA	193 (9.76%)	1.55 (1.13, 2.14)	0.007
Smoking			
No	1208 (61.07%)	Reference	
Yes	571 (28.87%)	1.07 (0.88, 1.31)	0.510
NA	199 (10.06%)	1.35 (0.99, 1.83)	0.057
Diabetes history			
No	1682 (85.04%)	Reference	
Yes	85 (4.30%)	3.22 (1.90, 5.46)	**< **0.01
NA	211 (10.67%)	1.50 (1.12, 2.02)	0.007
Hypertension history			
No	1463 (73.96%)	Reference	
Yes	304 (15.37%)	5.61 (4.05, 7.76)	**< **0.01
NA	211 (10.67%)	1.83 (1.36, 2.47)	**< **0.01

The bold values are used to highlight the association between the important indicators (Neutrophil, HDL-C and NHR) and atherosclerosis

*CI* Confidence interval; *OR* Odds ratio

**Table 3:** Multivariate analyses using different models to determine the relationship between the NHR and atherosclerosis

Variable	Crude model		Model I		Model II	
	OR (95%CI)	*P*	OR (95%CI)	*P*	OR (95%CI)	*P*
NHR	1.19 (1.11, 1.27)	**< **0.01	1.20 (1.11, 1.29)	**< **0.01	1.14 (1.06, 1.24)	0.001
NHR per-SD increase	1.29 (1.17, 1.43)	**< **0.01	1.30 (1.17, 1.46)	**< **0.01	1.22 (1.08, 1.37)	0.001
NHR groups						
Q1 (< 2.04)	Reference		Reference		Reference	
Q2 (≥ 2.04 ~ **< **2.72)	1.48 (1.15, 1.90)	0.002	1.45 (1.11, 1.91)	0.007	1.38 (1.03, 1.83)	0.029
Q3 (≥ 2.72 ~ **< **3.64)	1.76 (1.37, 2.27)	**< **0.01	1.86 (1.41, 2.47)	**< **0.01	1.64 (1.22, 2.20)	0.001
Q4 (≥ 3.64)	1.96 (1.52, 2.53)	**< **0.01	2.01 (1.51, 2.68)	**< **0.01	1.67 (1.22, 2.29)	0.001
*P* for trend	1.25 (1.15, 1.35)	**< **0.01	1.26 (1.15, 1.38)	**< **0.01	1.19 (1.08, 1.31)	0.001

The bold values are used to highlight the statistically significant association between NHR and atherosclerosis

Effect: Atherosclerosis, Crude model adjust for: None, Model I adjusted for: sex and age groups, Model II adjusted for: sex, age groups, BMI groups, HR, LDL-C groups, drinking, smoking, hypertension and diabetes history

*CI* Confidence interval; *OR* Odds ratio

**Fig. 2:**
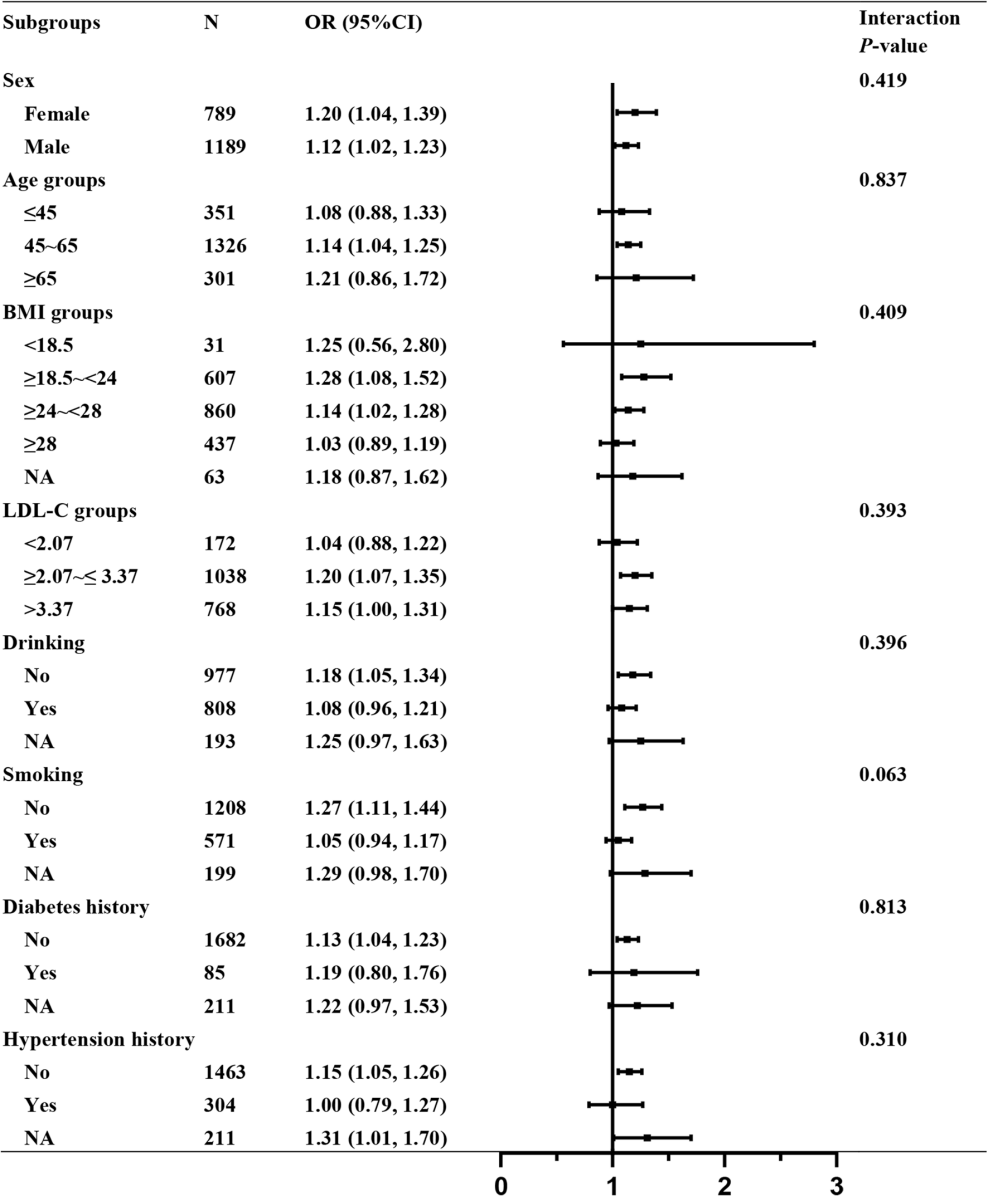
Subgroup analyses of the relationship between the NHR and atherosclerosis after adjustment for confounding factors. Adjusted for sex, age group, BMI group, HR, LDL-C group, drinking, smoking, hypertension and diabetes

We performed subgroup analyses to further explore the relationship between the NHR and atherosclerosis. As shown in Fig. 2, the positive relationship between the NHR and atherosclerosis revealed a highly consistent pattern. The NHR was positively associated with atherosclerosis in the female population (OR = 1.20, 95% CI: 1.04–1.39) as well as in the male population (OR = 1.12, 95% CI: 1.02–1.23). Thus, the positive relationship was consistent in the sex subgroups (interaction *P* = 0.419). Moreover, the positive relationship was consistent in the other subgroups including the age subgroups, BMI subgroups, LDL-C subgroups, drinking subgroups, smoking subgroups, diabetes subgroups and hypertension subgroups (OR ≥ 1, interaction *P* > 0.05).

### Nonlinear relationship between the NHR and atherosclerosis

**Fig. 3:**
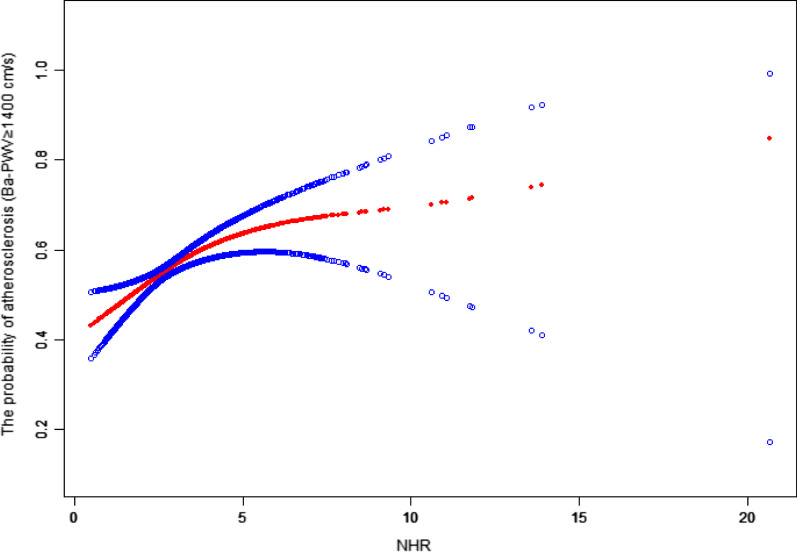
The nonlinear relationship between the NHR and atherosclerosis. A nonlinear association with a saturation effect was found between the MHR and Ba-PWV in a generalized additive model (GAM) (*P* < 0.01). Solid red line represents the smooth curve fit between variables. Blue bands represent the 95% confidence intervals from the fit. The model adjusted for sex, age group, BMI group, HR, LDL-C group, drinking, smoking, hypertension and diabetes

The study further applied a smoothing function analysis of the GAM to explore the potential nonlinear relationship between the NHR and atherosclerosis. The results revealed a non-linear relationship with a saturation effect between the NHR and atherosclerosis. Analysis of the threshold saturation effect showed that the threshold NHR value was 3.32. At values ≤ 3.32, the NHR was positively associated with atherosclerosis, but the association was not statistically significant beyond this threshold (Fig. 3; Table 4).

**Table 4:** Threshold effect analysis for the relationships between the NHR and atherosclerosis

Effects	Atherosclerosis (Ba-PWV ≥ 1400 cm/s)	
	OR (95%CI)	*P*
*Model*		
One-line	1.14 (1.06, 1.24)	0.001
*Model II*		
Turning point (T)	3.32	
Slope1: NHR**< **T	**1.35 (1.14, 1.59)**	**0.000**
Slope2: NHR ≥ T	1.04 (0.93, 1.16)	0.526
Slope2- Slope1	0.77 (0.61, 0.96)	0.022
A log likelihood ratio test	**0.024**	

## Discussion

The present study comprehensively evaluated the relationship between the NHR and atherosclerosis in a health examination population. The analysis of data from 1978 participants produced the following main findings. 1. The NHR was positively associated with atherosclerosis with strict control for confounding factors, and the positive association was consistent in different subgroups. 2. A nonlinear relationship with a saturation effect was demonstrated between the NHR and atherosclerosis; that is, NHR was positively associated with atherosclerosis at values ≤ 3.32, but the association was not statistically significant beyond 3.32.

Previous research has focused the association between NHR and cardiovascular and cerebrovascular disease outcomes. The NHR was shown to have good prognostic value in elderly patients with acute myocardial infarction and be associated with the severity and outcome of coronary artery disease [31, 32]. The utility of the NHR in cerebrovascular disease has also been demonstrated. Cheng et al. [33] found that high NHR values were associated with poor 3 month outcomes after intravenous thrombolysis in acute ischemic stroke patients, and Liu et al. [34] found that the NHR could be used to predict intracranial and extra cranial artery stenosis at different altitudes, especially at high altitudes. In addition, this study found that overweight or obese people accounted for a higher proportion of people in the Q4 group of NHR, and overweight and obese people have a higher risk of metabolic syndrome, chronic kidney and cardiovascular diseases. This may provide insight into the mechanism of the relationship between the NHR and metabolic diseases and kidney diseases [37]. However, these studies mainly focused the NHR and clinical diseases and not specifically on populations with early atherosclerosis. This may suggest that their findings might be biased, and therefore, cannot be extrapolated to the general population. The present study explored the relationship between the NHR and early atherosclerosis in a health examination population, which could be useful in providing early interventions for clinical disease.

It is well known that the BP level is closely related to arteriosclerosis, and therefore, accurate measurement of BP is very important. The Lancet Commission on Hypertension group summarized the current situation regarding the regulatory requirements and accuracy standards for BP measurement and then provided recommendations towards improving validation [36]. Our electronic sphygmomanometer has undergone strict quality control and meets the requirements for BP measuring devices [36]. Moreover, BP variability and blood pressure types are closely related to arteriosclerosis [38, 39]. Therefore, hypertension is an important confounding factor in the relationship between the NHR and arteriosclerosis. We adjusted for hypertension in our multivariate and smoothing function analyses and analyzed the relationship between the NHR and arteriosclerosis in two subgroups with and without hypertension. Thus, our results may comprehensively verify the relationship between the NHR and arteriosclerosis.

The mechanism underlying the relationship between the NHR and atherosclerosis remains unclear. Research suggests that inflammation and hyperlipidemia are central contributors to the development of atherosclerosis [13]. By combining the neutrophil count and HDL-C level, the NHR potentially reflects both inflammation and hyperlipidemia. Neutrophils are present at all stages of the development of atherosclerosis, and they promote the progression of atherosclerosis by promoting an inflammatory response and oxidative stress [29, 40]. Moreover, neutrophils also can produce vasoconstrictor substances and reactive oxygen species that cause vasospasm, which further leads to vascular smooth muscle proliferation, microalgae genesis, and atherosclerosis. In addition, neutrophil-induced aggregation and infiltration of inflammatory cells along with platelet aggregation result in vascular stenosis and blockage [41]. In contrast, HDL-C can prevent the development of atherosclerosis plaque by inhibiting the inflammatory reaction and inflammatory cell aggregation, protecting the vascular endothelium, and reversing cholesterol transport. In addition, elevated HDL-C levels can inhibit the production of various chemokines, hinder the oxidative stress-induced migration of macrophages and LDL-C transport, and counteract the effects of neutrophil-mediated inflammation and oxidative stress. Multiple epidemiological studies have reported a positive association between low levels of HDL-C and myocardial infarction, stroke, sudden death, and severe or premature coronary artery disease [42–44]. The combination of neutrophil count and HDL-C level into a dynamic comprehensive index captures both the enhancement of the tissue inflammatory response and the decrease in anti-inflammatory and antioxidant ability, which may reflect the relationship between inflammation and atherosclerosis.

A strength of the present study is that the NHR is a newly discovered index of inflammation, and the relationship between the NHR and atherosclerosis was explored for the first time in this study. Secondly, this study applied multiple approaches to strictly control for confounding factors, providing a certain degree of rigor to the analysis. Finally, we used the GAM to determine the nonlinear relationship between the NHR and atherosclerosis and identified a saturation effect in the curvilinear relationship, which will be important to consider in clinical practice.

This study also has some limitations. As a cross‐sectional study, the evidence for a causal relationship is relatively weak. Thus, the causal relationship needs to be further verified in prospective studies. In addition, the study excluded the population with coronary artery disease and cerebrovascular diseases. Therefore, the conclusion cannot be extrapolated to the people with coronary heart disease and cerebrovascular diseases. Moreover, this study was a single-center study, and thus, the results may be applicable to only a certain population and region. Furthermore, we did not collect data on statin therapy or other treatment, which may impact the real association between the NHR and atherosclerosis. Additionally, some indicators such as those related to work stress and psychological status, which might affect the prevalence of arteriosclerosis, were not collected. Finally, some data were missing in this study, which might have some impact on the results. Thus, further large-scale, multi-population prospective studies are needed to confirm and expand upon the findings of this study.

## Conclusion

A positive association was observed between the NHR and atherosclerosis. Furthermore, the relationship between the NHR and atherosclerosis was non-linear with a saturation effect, and the threshold NHR value for the saturation effect was 3.32. The NHR was positively associated with atherosclerosis before the threshold, but the association was not statistically significant beyond the threshold.

## Data Availability

The datasets generated during and/or analyzed during the current study are available from the corresponding author on reasonable request.

## Abbreviations

NHR: Neutrophil to high-density lipoprotein cholesterol ratio
Ba-PWV: Brachial-ankle pulse wave velocity
HDL-C: High-density lipoprotein cholesterol
ECG: Electrocardiogram
GAM: Generalized additive model
BMI: Body mass index
LDL-C: Low-density lipoprotein cholesterol
HR: Heart rate
OR: Odds ratio
CI: Confidence interval
